# P01.30. The role of pH in cancer

**DOI:** 10.1186/1472-6882-12-S1-P30

**Published:** 2012-06-12

**Authors:** I Robey

**Affiliations:** 1 University of Arizona, Tucson, USA

## Purpose

Malignant tumors are acidic. The extracellular pH of tumors ranges from 6.5-6.9 versus 7.2-7.4 in normal tissue. Acid-producing tumors are more invasive and exhibit a greater metastatic potential. Tumor acidity is toxic to the surrounding tissue, induces the breakdown of the basement membrane around the tumor, and increases expression and activity of metastatic effectors. Therefore, it is thought that tumor acidity is a selective advantage in promoting cancer progression.

## Methods

A corollary to these observations asserts that reversal or buffering of tumor acidity could inhibit tumor invasion or metastases. Buffering of tumor acidity can be achieved through systemic administration of a natural alkaline buffer. This has been demonstrated in tumor bearing mice using sodium bicarbonate drinking water.

## Results

Systematic bicarbonate selectively raises the extracellular pH (pHe) surrounding tumors from acidic levels (6.8) to physiological levels (7.2). Mice bearing breast and prostate tumors had reduced metastases and significantly improved survival compared to untreated tumor bearing mice. The reduced activity of invasive mechanisms such as cathepsin B and significantly lower numbers of circulating tumor cells in the blood stream of mice maintained on oral bicarbonate supports the premise that buffering tumor acidity slows acid-mediated tumor invasion. The findings from these studies also indicate that chronic administration of bicarbonate does not affect the serum pH of mice or cause measurable acute or chronic adverse health effects.

## Conclusion

Manipulation of the tumor microenvironment is a potential avenue for therapeutic approaches in cancer care. The use of natural alkaline compounds against cancer is relatively non-toxic and may provide benefit in specific cases. Sodium bicarbonate, for example, may be useful as an adjunctive therapy with other buffers or agents that reduce/inhibit tumor acidity. Additionally, there is potential utility for systemic bicarbonate as a safe method for alkalinizing highly protonated chemotherapeutics against acidic tumors.

